# Untargeted Liquid Chromatography–High-Resolution Mass Spectrometry Metabolomic Investigation Reveals Altered Lipid Content in *Leishmania infantum* Lacking Lipid Droplet Protein Kinase

**DOI:** 10.3390/tropicalmed9090208

**Published:** 2024-09-10

**Authors:** Juliana Martins Ribeiro, Gisele André Baptista Canuto, Alisson Samuel Portes Caldeira, Ezequias Pessoa de Siqueira, Carlos Leomar Zani, Silvane Maria Fonseca Murta, Tânia Maria de Almeida Alves

**Affiliations:** 1 Grupo Genômica Funcional de Parasitos, Instituto René Rachou, Fundação Oswaldo Cruz, Belo Horizonte 30190-002, Minas Gerais, Brazil; juliana.mribeiro@fiocruz.br; 2 Departamento de Química Analítica, Instituto de Química, Universidade Federal da Bahia, Salvador 40170-115, Bahia, Brazil; gisele.canuto@ufba.br; 3 Grupo Química de Produtos Naturais Bioativos, Instituto René Rachou, Fundação Oswaldo Cruz, Belo Horizonte 30190-002, Minas Gerais, Brazil; alisson.caldeira@fiocruz.br (A.S.P.C.); ezequias.siqueira@fiocruz.br (E.P.d.S.); carlos.zani@fiocruz.br (C.L.Z.)

**Keywords:** lipid droplet protein kinase, *L. infantum*, untargeted metabolomics, liquid chromatography–high-resolution mass spectrometry (LC-HRMS)

## Abstract

Leishmaniasis is a complex disease caused by different species of *Leishmania*. To date, no vaccine for humans or ideal therapy has been developed owing to the limited efficacy and toxicity of available drugs, as well as the emergence of resistant strains. Therefore, it is necessary to identify novel therapeutic targets and discover therapeutic options for leishmaniasis. In this study, we evaluated the impact of deleting the lipid droplet protein kinase (LDK) enzyme in *Leishmania infantum* using an untargeted metabolomics approach performed using liquid chromatography and high-resolution mass spectrometry. LDK is involved in lipid droplet biogenesis in trypanosomatids. Thirty-nine lipid metabolites altered in the stationary and logarithmic growth phases were noted and classified into five classes: (1) sterols, (2) fatty and conjugated acids, (3) ceramides, (4) glycerophosphocholine and its derivatives, and (5) glycerophosphoethanolamine and its derivatives. Our data demonstrated that glycerophosphocholine and its derivatives were the most affected after LDK deletion, suggesting that the absence of this enzyme promotes the remodeling of lipid composition in *L. infantum*, thus contributing to a better understanding of the function of LDK in this parasite.

## 1. Introduction

Leishmaniasis is a neglected tropical disease caused by obligate intracellular protozoan parasites of the *Leishmania* genus [1]. It affects vulnerable populations in 98 countries in the tropical and subtropical areas of the Americas, Africa, Asia, Europe, and Oceania. It is estimated that more than one billion individuals are at risk of infection, with an incidence of 0.7 to 1 million new cases per year [2]. It is transmitted to humans through the bite of infected female sandflies and can manifest in two main clinical forms: tegumentary leishmaniasis (TL) (characterized by skin and/or mucous lesions) and visceral leishmaniasis (VL) (affects internal organs, especially the liver, spleen, and bone marrow). VL is the most severe manifestation of the disease in humans and is fatal if left untreated [3,4]. It is caused by *L. donovani* in Asia and Africa and *L. infantum* in the Mediterranean Basin, Middle East, Central Asia, South America, and Central America [3,4].

Currently, no vaccine is available for humans, and the lack of efficient vector control initiatives highlights the need for effective chemotherapeutic interventions to control this disease [5,6,7]. There is no ideal therapy for leishmaniasis, and the available options (miltefosine, pentavalent antimonials, and amphotericin B formulations) have limited efficacy, high toxicity, and are hampered by the emergence of resistant strains [5,7].

Therefore, the search for novel drug targets of parasites is imperative for developing new medicines to treat leishmaniasis. A potential candidate is a lipid droplet protein kinase (LDK), which is involved in the biogenesis of lipid droplets (LDs) in trypanosomatids [8,9]. Lipid droplets are cellular organelles primarily composed of neutral lipids such as triglycerides and cholesterol esters surrounded by a phospholipid monolayer and associated proteins. These structures play a crucial role in lipid metabolism, storage, and signaling within cells [10,11,12] and are cellular organelles present in all cell types and organisms, including protozoan parasites [11]. LDs are involved in several cellular processes, including lipogenesis, lipolysis, lipid trafficking, nutrient storage, and energy metabolism [10,12].

LD biogenesis and maintenance in trypanosomatids have not yet been well elucidated, but studies have highlighted the importance of LDK in these processes in both *Trypanosoma brucei* [8] and *L. infantum* [9]. Recently, we deleted the LDK enzyme in *L. infantum* and observed a reduction in the number of LDs in the stationary phase of culture. Additionally, the deletion of LDK decreases the intracellular proliferation capacity of parasites and makes them resistant to trivalent antimony [9]. Metabolomics has emerged as an important strategy for understanding the metabolic changes that occur after LDK deletion in these parasites and may contribute to the observed phenotype.

By providing a comprehensive view of the intricate metabolic networks that govern the survival and pathogenicity of parasites [13,14], metabolomic studies of *Leishmania* parasites can contribute significantly to our understanding of host–parasite interactions and the development of diagnostic and therapeutic strategies for leishmaniasis. This study aimed to investigate the relevant metabolic differences between *L. infantum LDK*-knockout and its parental line (Cas9) using an untargeted metabolomics approach involving liquid chromatography–high-resolution mass spectrometry (LC-HRMS).

## 2. Materials and Methods

### 2.1. Parasite Culture Conditions

Promastigotes of *L. infantum* RPV (MHOM/BR/2002/LPC-RPV) constitutively expressing Cas9 and knockout for the LDK enzyme (ΔLDK), previously obtained by CRISPR/Cas9 [9], were maintained at 26 °C in M199 medium (Gibco, Waltham, MA, USA) supplemented with 40 mM HEPES (pH 7.4), 5 μg/mL hemin, 2 μg/mL biopterin, 1 μg/mL biotin, 2 mM L-glutamine, 500 U/mL penicillin, 50 μg/mL streptomycin, and 10% inactivated fetal bovine serum. To the cultures of ΔLDK parasites, 40 µg/mL neomycin and 30 µg/mL blasticidin were added. Culture maintenance was carried out twice a week, and 1 × 10^6^ parasites were inoculated into 5 mL of the culture medium.

### 2.2. Sample Collection

Cell collection was performed as previously described by Canuto et al. [15]. Briefly, the parasites were cultured, inoculated at 1 × 10^6^ parasites/mL, and harvested at 48 h (logarithmic phase) and 96 h (stationary phase). Three independent flasks (biological replicates) were prepared for each *L. infantum* culture (Cas9 and ΔLDK) and for each growth phase (logarithmic and stationary). After the growth period, the samples were immediately quenched on dry ice and kept cold throughout the procedure. The culture medium was removed by centrifugation at 1000× *g* for 10 min at 4 °C. The supernatant was discarded and the parasites were washed twice with PBS. Parasites were counted using a Z1 Coulter Particle Counter (Beckman Coulter, Brea, CA, USA), and 1 × 10^8^ parasites were placed in microtubes and centrifuged (1000× *g* for 10 min at 4 °C), after which the supernatant was removed. The sample pellets were maintained at −80 °C until metabolite extraction and biomass determination.

### 2.3. Extraction of Intracellular Metabolites

For LC-HRMS analysis, metabolites were extracted with 500 µL of pure cold methanol (HPLC grade), followed by lysis using an ultrasound probe (VC-750, Sonics vibra-cell—Sonics and Materials, Newton, MA, USA) for 1 min at an output power of 30%, 10 W. Centrifugation was performed (10,000× *g* for 10 min), and 200 µL of each supernatant were collected and mixed to compose the quality control (QC) pool. Another 200 µL of the supernatant from each sample were transferred to vials. A blank sample was prepared, and the extraction solution was subjected to the same extraction conditions. All samples, QCs, and blanks were maintained at −20 °C overnight until analysis. The samples analyzed using LC-HRMS were randomly distributed.

### 2.4. LC-HRMS Equipment

LC-HRMS analyses were performed on a Nexera UHPLC system (Shimadzu, Corporation, Kyoto, Japan) coupled with a MaXis high-resolution ESI-QToF mass spectrometer (Bruker, Billerica, MA, USA) controlled by the Compass 1.7 software package (Bruker). Fifteen microliters of each sample extract were injected into an ACQUITY UPLC BEH C18 column (130 Å, 1.7 μm, 2.1 mm × 75 mm) (Waters Corporation, Milford, MA, USA) maintained at 40 °C at a flow rate of 400 μL/min. Gradient elution was performed using mobile phases A and B (0.1% formic acid in deionized water and acetonitrile, respectively) starting at 2% B for 5 min, followed by a linear gradient to 100% B for 25 min and holding at 100% B for 5 min. Mass spectra were acquired in the positive ion mode at a spectral rate of 2 Hz. The ion-source parameters were set to 500 V end-plate offset, 4500 V capillary voltage, 3.0 bar nebulizer pressure, and 8 L/min and 200 °C dry gas flow and temperature, respectively. The fragment spectra of the QC and standard samples were acquired via data-dependent acquisition (DDA) using a collision energy of between 15 and 60 eV. To increase the number of QC fragment spectra, two additional QC runs were performed, excluding the previously selected parent ions. Mass calibration was achieved by an initial ion-source infusion of 25 µL of calibrant solution (1 mmol/L sodium formate in 50% 2-propanol) and post-acquisition recalibration of the raw data.

### 2.5. Data Processing and Statistical Analysis

Raw data were converted to *. mzML using the ProteoWizard software (version 3) for processing in the XCMS software package (version 3.8.2) running on the RStudio platform (version 3.6.3). Data extraction was performed using the “centWave” method with the following parameters: maximum and minimum peak width of 3 and 30, respectively; maximal tolerated *m*/*z* deviation in consecutive scans (ppm) of 5, signal-to-noise cutoff (snthresh) of 2, bandwidth (bw) of 10, and width of overlapping *m*/*z* slices (mzwid) of 0.025. Nonlinear alignment for the default “retcor” method was applied for retention time alignment, and the “fillPeaks” tool was used to remove missing values.

Statistical analyses were performed using MetaboAnalyst 6.0 platform (https://www.metaboanalyst.ca/, accessed on 2 June 2024). The data were filtered by removing molecular features with an RSD of >30% in the QC samples, followed by median normalization, log transformation, and Pareto scaling. Multivariate analysis using principal component analysis (PCA) was performed to inspect the samples, and QC clustering was performed to assess instrumental stability. Partial least squares discriminant analysis (PLS-DA) and orthogonal least squares discriminant analysis (OPLS-DA) were used to identify discriminants by variable importance in projection (VIP) score > 1.0 between sample groups. Volcano plots and false discovery rates (FDRs < 0.05) were used to identify the discriminants.

### 2.6. Metabolite Annotation, Enrichment, and Network Analyses

Metabolite annotation was performed at significant *m*/*z* values by searching the CEU Mass Mediator platform (http://ceumass.eps.uspceu.es/, accessed on 1 November 2023) using [M + H]^+^, [M + Na]^+^, and [M + 2H]^2+^ as adducts, with a maximum error of 5 ppm. Annotation was carried out with DDA data by searching the MS/MS spectra in MetaboScape 4.0 and MSDial (version 4.9.221218) software using the MassBank (MS/MS positive) database. Superclass and network enrichment analyses were performed using MetaboAnalyst 6.0 and LINEX^2^ (Lipid Network Explorer, https://exbio.wzw.tum.de/linex/, accessed on 23 February 2024), respectively. A full lipid class network with node size scaled by fold change was used to compare the ΔLKD and Cas9 groups.

## 3. Results

### 3.1. Changes in the Metabolic Profiles of L. infantum LDK between the Logarithmic and Stationary Phases of Growth

**Figure 1:**
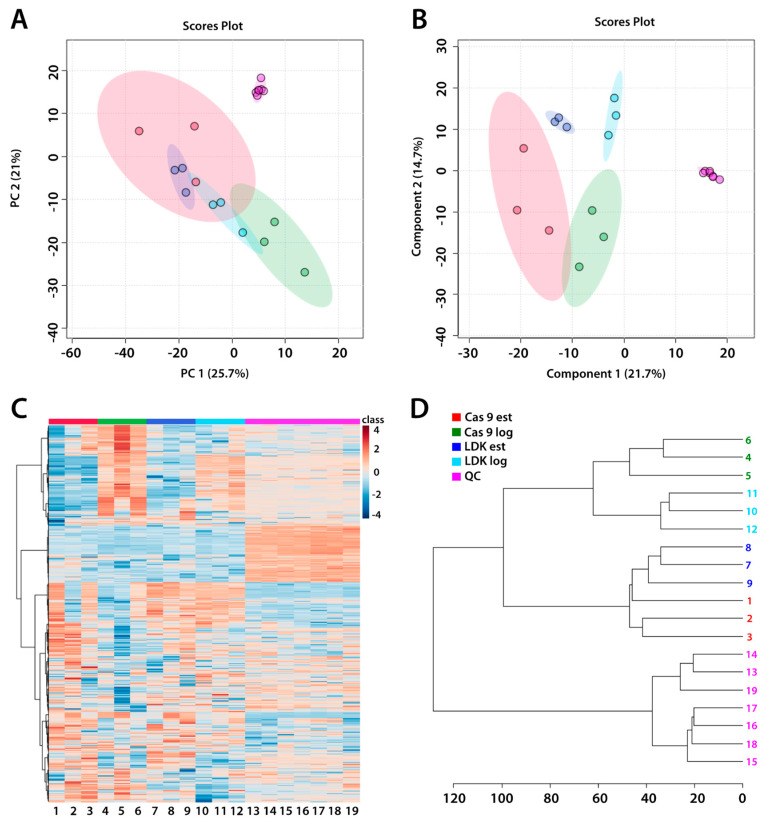
(**A**) PCA model of all samples and Pareto-scaled QCs, (**B**) PLS-DA model of all samples and Pareto-scaled QCs (quality parameters: R^2^ = 0.96 and Q^2^ = 0.64), (**C**) heat map of the entire metabolome dataset, and (**D**) HCA of the sample groups and QCs. Labels: pink, quality control (QC); red, Cas9 stationary phase; green, Cas9 logarithmic phase; blue, ΔLDK stationary phase; and cyan, ΔLDK logarithmic phase.

To understand the function of the LDK enzyme in *L. infantum*, the gene encoding this enzyme was previously deleted using CRISPR/Cas9 [9], and the parasites were subjected to untargeted metabolomic analysis using LC-HRMS. Samples of knockout parasites were obtained from the logarithmic and stationary growth phases and compared with those of the control parasites (Cas9), which were extracted in parallel under the same conditions. To evaluate the instrumental stability during analytical sequencing, as well as sample group clustering, we built multivariate models (PCA and PLS-DA, Figure 1). The models showed excellent clustering of samples and QCs samples, confirming the quality of the analytical data and the differences among the metabolic profiles. Additionally, hierarchical cluster analysis (HCA) and the heat map (Figure 1) revealed biological differences. The PLS-DA model showed good-quality parameters (R^2^ > 0.9 and Q^2^ > 0.6). The model was validated using permutation tests, with a *p*-value of 0.001 for 1000 permutations.

To identify discriminants, PLS-DA and OPLS-DA models were built to compare the stationary and logarithmic phases of LDK vs. Cas 9 (Figure S1 in Supplementary Materials). A VIP score (>1.0) was used to identify significant molecular features. Multivariate analyses revealed 1674 and 386 molecular features with VIP scores > 1.0 for stationary and logarithmic comparisons, respectively. In addition, the volcanic plot (Figure S2 in the Supplementary Materials) complemented the significant *m*/*z* values.

### 3.2. LDK Deletion Altered the Lipid Profile of L. infantum

After searching public databases and evaluating the MS/MS fragmentation patterns with different databases, 39 metabolites and lipids that were altered in the stationary and logarithmic phases were annotated. Table S1 (Supplementary Materials) provides the chemical information, MS/MS spectra, mass error, and annotation levels (according to the Metabolomics Standards Initiative) for all metabolites and annotated compounds. These alterations, determined by the fold change (FC), calculated by the ratio of ΔLDK/Cas9 control parasites, are presented in Table 1 and categorized by classes. As expected from the type of chromatographic column used (with a C18 stationary phase), compounds with nonpolar characteristics were separated and detected using mass spectrometry. Although metabolites belonging to different chemical classes were detected, significant changes were observed in their lipid profiles. We classified the altered annotated lipids into five classes: (1) sterols, (2) fatty acids and conjugates, (3) ceramides, (4) glycerophosphocholine and its derivatives, and (5) glycerophosphoethanolamine and its derivatives.

Interestingly, the data demonstrated that glycerophosphocholine and its derivatives (glycerophospholipids) were the most significantly affected after LDK deletion, as this class presented the largest quantity of metabolites identified (Table 1). This observation was confirmed by the enrichment analysis of altered metabolic pathways, whose *p* values for the stationary and logarithmic phase comparisons were 1.27 × 10^−33^ and 6.27 × 10^−27^, respectively. We observed that all identified lysophosphatidylcholine (LPC) decreased in the logarithmic phase and increased in the stationary phase in the ΔLDK knockout parasites compared to the control Cas9 parasites, except for LPC18:4, which also decreased in the logarithmic phase (Table 1). All identified phosphatidylcholines (PCs) were reduced in the log phase, except for PCs 36:2, 38:9, 40:9, and 42:9, which increased in this phase. In contrast, in the stationary phase, the PCs were more elevated in the ΔLDK knockout parasites than in the control Cas9 parasites, except for the PCs 32:2 and 40:8, which presented reductions (Table 1).

The absence of LDK in *L. infantum* parasites promoted a significant reduction in ergosta-5,7,22,24(28)-tetraen-3beta-ol levels during the stationary phase, similar to the metabolites octadecatetraenoic acid and methyl 5S,6R-epoxy-7-eicosynoate during the logarithmic phase. The levels of cer(34:1;O2) from the sphingolipid and ceramide classes increased during the log and stationary phases of growth (Table 1). The metabolites lysophosphatidylethanolamines (LPEs) 18:1 and 18:2 were also identified, with the latter being reduced in ΔLDK knockout parasites in both growth phases evaluated (Table 1).

### 3.3. Metabolism of Glycerophospholipids Was the Main Pathway Impacted by LDK Deletion in L. infantum

**Figure 2:**
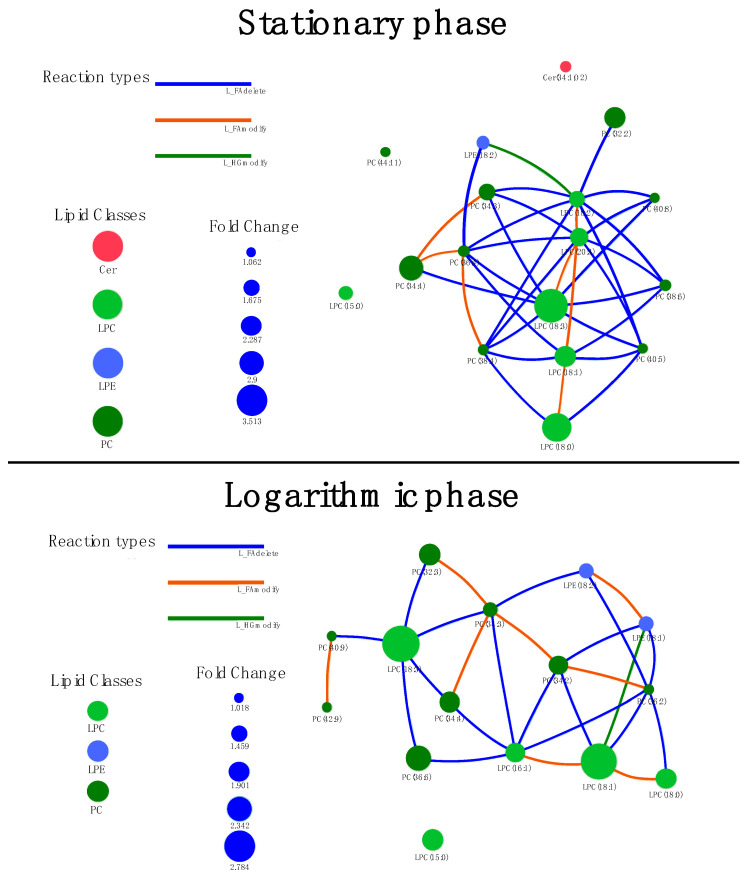
Full lipid network with node size scaled by fold change for comparison between *L. infantum* ΔLDK and Cas9. (**Up**) Stationary phase and (**down**) logarithm phase. Lipids are colored by class, and edge colors indicate reaction types connecting two nodes. Larger nodes correspond to greater abundance in the ΔLDK group, and small nodes correspond to greater abundance in the Cas9 group.

Network analysis of the identified lipid classes, in which the nodes are based on fold change (ΔLDK vs. Cas9), was performed for both the stationary and the logarithmic phase. Figure 2 presents the results obtained using the LINEX platform.

There was a significant correlation between the LPE 18:2 → 18:1 lipid node. In the stationary phase, no significant correlation was detected between the lipid nodes (assessed by FDR).

Network analysis verified that phosphatidylcholines (PCs) and lysophosphocholines (LPCs) were typically more abundant in *L. infantum* ΔLDK in both growth phases evaluated. We also demonstrated, through enrichment analysis by MetaboAnalyst 6.0, that ΔLDK knockout had the greatest impact on the glycerophosphocholine class of lipids, demonstrating high significance both in the logarithmic phase (FDR = 6.27 × 10^−27^) and in the stationary phase (FDR = 1.27 × 10^−33^).

## 4. Discussion

In this study, we performed untargeted metabolomics using LC-HRMS to address the differences between *L. infantum* parasite knockouts of the LDK enzyme and parental control parasites during the log and stationary phases of growth. Table 1 shows the most significant changes in lipid levels and their derivatives. This result was expected because the untargeted analysis was performed on a reversed-phase C18 column, which mainly retained substances with more nonpolar characteristics. LDK is associated with the biogenesis and maintenance of LDs in trypanosomatids [8,9]. Lipids are the main constituents of cell membranes and are important for maintaining the structural and functional integrity of cells. They are important signaling mediators involved in several aspects of signal transduction [16].

We observed, according to the identified PCs, a reduction in the log phase in the ΔLDK parasites compared to that in the control parasites (Cas9), except for PCs 36:2, 39:8, 40:9, and 42:9. In the stationary phase, we observed the opposite trend, with an increase in most identified PCs, except for PCs 32:2, 40:7, and 40:8. Glycerophospholipids (a class of phospholipids), are important in the development of *Leishmania* parasites. The two main classes of glycerophospholipids, important components of the cell membrane, are phosphatidylethanolamine (PE) and PC [17,18]. PE accounts for approximately 10% of the total lipids in *Leishmania*, and PC is the most abundant, accounting for approximately 30–40% of the total lipids [18,19]. In *Leishmania*, PE promotes membrane fusion, in addition to being necessary for the synthesis of GPI-anchored proteins, providing ethanolamine phosphate, which binds proteins to glycan anchors. PE contributes to autophagosome formation during differentiation and starvation [19]. It can be synthesized in three different ways: (1) through the Kennedy reaction (ethanolamine ⇒ ethanolamine phosphate ⇒ CDP-ethanolamine ⇒ PE), (2) by decarboxylation of phospholipids that occur in the mitochondria or Golgi complex, and (3) by the exchange of bases between PE, PC, and phospholipids [17].

In contrast, phosphatidylcholine is a precursor of important signaling molecules and metabolic intermediates, including lysophosphatidylcholine, phosphatidic acid, diacylglycerol, and free fatty acids [19]. PCs are synthesized *de novo* via the Kennedy reaction (choline ⇒ choline-phosphate ⇒ CDP-choline ⇒ PC), by methylation of PE carried out by one or more methyltransferases, or through acquisition from host or culture medium lipids [18,19]. PCs are composed of 1,2-diacyl and 1-lyso-2-acyl unsaturated long-chain fatty acids. Although their functions are not yet well understood, polyunsaturated fatty acids can modulate the physiology of the membrane by reducing its melting point and conferring resistance to host-derived oxidants [17]. Long-chain fatty acids (LCFAs) have recently been suggested to function as energy sources for these parasites [20].

Our data demonstrated that there was a decrease in lysophosphatidylethanolamine (LPE) content in both the logarithmic and the stationary phase of the ΔLDK parasites compared to that in the control (CAS9) parasites (Table 1). Interestingly, we showed that the levels of lysophosphatidylcholines (LPCs), but not LPC (18:4), decreased in the logarithmic phase but increased in the stationary phase. LPEs and LPCs are generated by the hydrolysis of PEs and PCs, respectively, by phospholipase A2 [21]. Lysophospholipids are the intermediate metabolites of glycerophospholipid metabolism. At low levels, they are less harmful to lipid bilayers, whereas increased levels typically occur during cellular stress. Although the biological functions of LPEs have not yet been elucidated, they are involved in cell proliferation and differentiation, oxidative stress, apoptosis induction, and proinflammation [21]. In *Leishmania*, LPCs promote parasite proliferation by inhibiting oxidative and nitrosative stress and by supporting arginase-1 activity. The pro-inflammatory effect of LPCs depends on the length of the acyl chain and the degree of saturation. Saturated LPCs such as LPC 16:0 are potent inflammatory mediators that can induce plasma leakage, immune cell migration, and proinflammatory cytokine release. In contrast, polyunsaturated LPCs, including 20:4 and 22:6 LPC, can act as anti-inflammatory lipid mediators and inhibit inflammation induced by saturated LPCs. They also reduce plasma leakage and the activation of inflammatory cells, inhibit the production of inflammatory mediators, and increase the production of anti-inflammatory factors [21].

In our experiments, Cer(34:1;O2) levels were stable during both growth phases (FC = 1.03). Spisulosine was identified only in the log phase (FC = 1.20). The concentration of spilusoline in BPK026 SbIII-resistant *Leishmania donovani* appeared to be half of that detected in the wild type [22]. Sphingolipids (SLs) constitute another important group of membrane components in eukaryotes involved in signaling processes including apoptosis, cell recognition, growth, and differentiation [17]. *Leishmania* parasites do not synthesize sphingomyelin, and most SL compounds are non-glycolyzed inositol phosphorylceramide and ceramide, which consist of sphingosine d18:1 and fatty acids C24:1 and C16:0. In particular, ceramide (generated by *de novo* synthesis and/or degradation of other SLs) is an important second messenger capable of activating different enzymes, such as PP1 and PP2A phosphatases, protein kinase Czeta, and cathepsin D [17]. In *L. donovani*, there is an increase in the *de novo* synthesis of ceramides in macrophages, which favors the establishment of infection [23]. In *L. major*, ceramides protect against membrane damage [24].

SLs are composed of a sphingoid base of 1–20 carbon atoms and amino, hydroxyl, and methyl groups. The main sphingoid bases in eukaryotes are sphingosine, sphinganine, and phytosphingosine; however, more than 60 different types of sphingoid bases have been identified. These molecules generate ceramides when N-acylated at the free amino group at C-2 by fatty acid [17]. Sphingoid bases are important for intracellular signaling, apoptosis, regulation of cell growth and differentiation, and the modulation of immune responses [25]. Okundaye et al. [26] demonstrated that sphingoid base metabolism regulates vital processes that ensure the viability of *Leishmania* parasites. Additionally, it is important to provide ethanolamine phosphate for synthesizing PEs and PCs. Zhang et al. [25] showed that in *L. major*, sphingoid bases are required in the stationary phase and not in the log phase.

Among sterols, ergosterol is dominant in the *Leishmania* membrane [17] and is important for maintaining its structure and function [27]. Sterol C-24 reductase catalyzes the conversion of ergostructetraenol to ergosterol in the final step of ergosterol biosynthesis. The absence of this enzyme is associated with a lack of ergosterol and leads to the accumulation of its substrate, ergosta-5,7,22,24(28)-tetraen-3β-ol [28]. In our experiments, the level of this steroid decreased (FC = 0.84) in the stationary growth phase of delta LDK parasites compared to that in the control (Cas9) parasites. Yao and Wilson [29] demonstrated that a change in sterol content occurs during metacyclogenesis in *L. infantum*, suggesting an association between sterol content and parasite virulence. Other studies have suggested that the inhibition of ergosterol biosynthesis decreases cell viability [30].

We identified the putative fatty acids octadecatetraenoic acid and methyl 5S,6R-epoxy-7-eicosynoate only in the logarithmic phase. Trypanosomatids can synthesize fatty acids de novo or via uptake from the host. They are essential for the growth and survival of parasites during the life cycle of *T. brucei*, *T. cruzi*, and *Leishmania*, because oxidized fatty acids are the main source of energy [31].

The LDK enzyme is associated with the production of LDs in trypanosomatids [8], and a recent study published by our group revealed that deletion of this enzyme reduces the number of LDs in the stationary phase of growth. Furthermore, LDK is important for the biogenesis of LDs and maintaining normal quantities of these organelles in *L. infantum* [9]. The structure of LDs comprises a phospholipid monolayer, commonly PC, which surrounds a core composed of neutral lipids, mainly triacylglycerols, and sterol esters [12]. Under the evaluated conditions, our data demonstrated that LDK was intrinsically related to lipid metabolism in *L. infantum*, as different lipid classes were affected by the deletion of this enzyme.

The deletion of LDK in *L. infantum* showed that LPE 18:2 and LPE 18:1 were correlated in the logarithmic phase. As previously described, LPE is produced through the hydrolysis of PE by phospholipase A2 [21], but its function in *Leishmania* parasites has not yet been elucidated. Zuffrerey et al. [32] detected LPE 18:1 and 18:2 in *L. major* but in small quantities. Our data suggest that deletion of LDK may have altered the metabolism of this class of lipids, promoting deregulation in the logarithmic phase. We also demonstrated that glycerophospholipid metabolism was most affected by the absence of LDK. These findings suggest that glycerophospholipids are important for the development of *L. infantum* and that the exclusion of LDK affects this pathway either by altering the biogenesis of LDs, as glycerophospholipids are the main components [33], or through the structure of *Leishmania* as a whole, as these classes of lipids are important components of the cell membrane [17,18]. Further studies must be conducted to verify whether other enzymes and pathways are affected by LDK deletion.

## 5. Conclusions

According to our previous results, LDK deletion reduced lipid droplet production in *L. infantum* during the stationary phase [9]. In addition, LDK knockout mutants showed a decreased ability to maintain infection in macrophages and were more resistant to trivalent antimony [9]. Interestingly, our data presented here show that the deletion of LDK promoted the remodeling of lipid composition in *L. infantum* so that the parasites could develop and produce LDs without this enzyme by compensating through other pathways; however, this has yet to be determined. Future studies are needed to expand metabolic coverage, such as using complementary analytical platforms and confirming annotated metabolites using authentic analytical standards. These data provide novel insights into a better understanding of the functions of LDK in *L. Infantum.*

## Figures and Tables

**Table 1:** ΔLDK vs. Cas9 parasites.

Metabolites	Log Phase (Fold Change)	Stationary Phase (Fold Change)
**Sterols**		
Ergosta-5,7,22,24(28)-tetraen-3beta-ol		↓ (0.84)
**Fatty Acids and Conjugates**		
Octadecatetraenoic acid	↓ (0.63)	
Methyl 5S,6R-epoxy-7-eicosynoate	↓ (0.72)	
**Sphingolipids and Ceramide**		
Spisulosine	↑ (1.20)	
Ceramide—Cer(34:1;O2)		↑ (1.03)
**Glycerophosphocholine and derivatives**		
Lysophosphatidylcholine—LPC (15:0)	↓ (0.65)	
Lysophosphatidylcholine—LPC (15:1)	↓ (0.57)	↑ (1.54)
Lysophosphatidylcholine—LPC (16:1)	↓ (0.68)	
Lysophosphatidylcholine—LPC (18:0)	↓ (0.67/0.42 *)	↑ (3.06/4.26 *)
Lysophosphatidylcholine—LPC (18:1)	↓ (0.42)	↑ (1.24/2.28 *)
Lysophosphatidylcholine—LPC (18:2)		↑ (1.79)
Lysophosphatidylcholine—LPC (18:3)	↓ (0.43)	↑ (3.64)
Lysophosphatidylcholine—LPC (18:4)		↓ (0.63)
Lysophosphatidylcholine—LPC (20:3)		↑ (2.09)
Lysophosphatidylcholine—LPC (20:4)	↓ (0.42)	
Lysophosphatidylcholine—LPC (22:6)		↑ (2.09)
Phosphatidylcholine—PC (32:2)		↓ (0.78)
Phosphatidylcholine—PC (32:3)	↓ (0.66)	
Phosphatidylcholine—PC (33:5)	↓ (0.51/0.55 *)	
Phosphatidylcholine—PC (34:2)	↓ (0.70)	
Phosphatidylcholine—PC (34:3)	↓ (0.84)	↑ (1.16)
Phosphatidylcholine—PC (34:4)	↓ (0.67)	↑ (1.88)
Phosphatidylcholine—PC (34:6)	↓ (0.84/0.55 *)	
Phosphatidylcholine—PC (36:7)	↓ (0.73)	↑ (1.20)
Phosphatidylcholine—PC (38:4)		↑ (1.04)
Phosphatidylcholine—PC (38:6)		↑ (1.63)
Phosphatidylcholine—PC (38:7)		↑ (1.05)
Phosphatidylcholine—PC (38:9)	↑ (1.21)	↑ (1.04)
Phosphatidylcholine—PC (40:5)		↑ (1.05)
Phosphatidylcholine—PC (40:8)		↓ (0.79/0.83 *)
Phosphatidylcholine—PC (40:9)	↑ (1.14)	↑ (1.63)
Phosphatidylcholine—PC (42:9)	↑ (1.19)	
Phosphatidylcholine—PC (42:10)		↑ (1.40)
Phosphatidylcholine—PC (44:11)		↑ (2.70)
**Glycerophosphoethanolamine and derivatives**		
Lysophosphatidylethanolamine—LPE (18:1)	↓ (0.57)	
Lysophosphatidylethanolamine—LPE (18:2)	↓ (0.85)	↓ (0.84)

Legend: (↓) decreased in ΔLDK parasites and (↑) increased in ΔLDK parasites; * the first fold change refers to the hydrogen adduct, and only the second fold change refers to the sodium adduct. The fold change was obtained with the MetaboAnalyst 6.0 platform and was calculated as the ratio of the average signal from the first group to the average signal from the second group.

## Data Availability

The original contributions presented in the study are included in the article/Supplementary Materials, further inquiries can be directed to the corresponding authors.

## Supplementary Materials

The following supporting information can be downloaded at: https://www.mdpi.com/article/10.3390/tropicalmed9090208/s1, Figure S1: OPLS-DA and PLS-DA models for ΔLDK vs. Cas9 *L. infantum*; Figure S2: Volcano plots of molecular features altered in ΔLDK vs. Cas9 *L. infantum*. Table S1: Metabolite annotation and level of confidence according to the MSI (Metabolomics Standards Initiative).
