# Supplementary material for: Untargeted Liquid Chromatography–High-Resolution Mass Spectrometry Metabolomic Investigation Reveals Altered Lipid Content in Leishmania infantum Lacking Lipid Droplet Protein Kinase
Source: Trop Med Infect Dis. 2024 Sep 10;9(9):208. doi: 10.3390/tropicalmed9090208 (PMC11435790; doi:10.3390/tropicalmed9090208)
Supplement: Supplementary file 1 [file tropicalmed-09-00208-s001.zip › tropicalmed-3090547-Supplementary.pdf]

Supplementary Materials

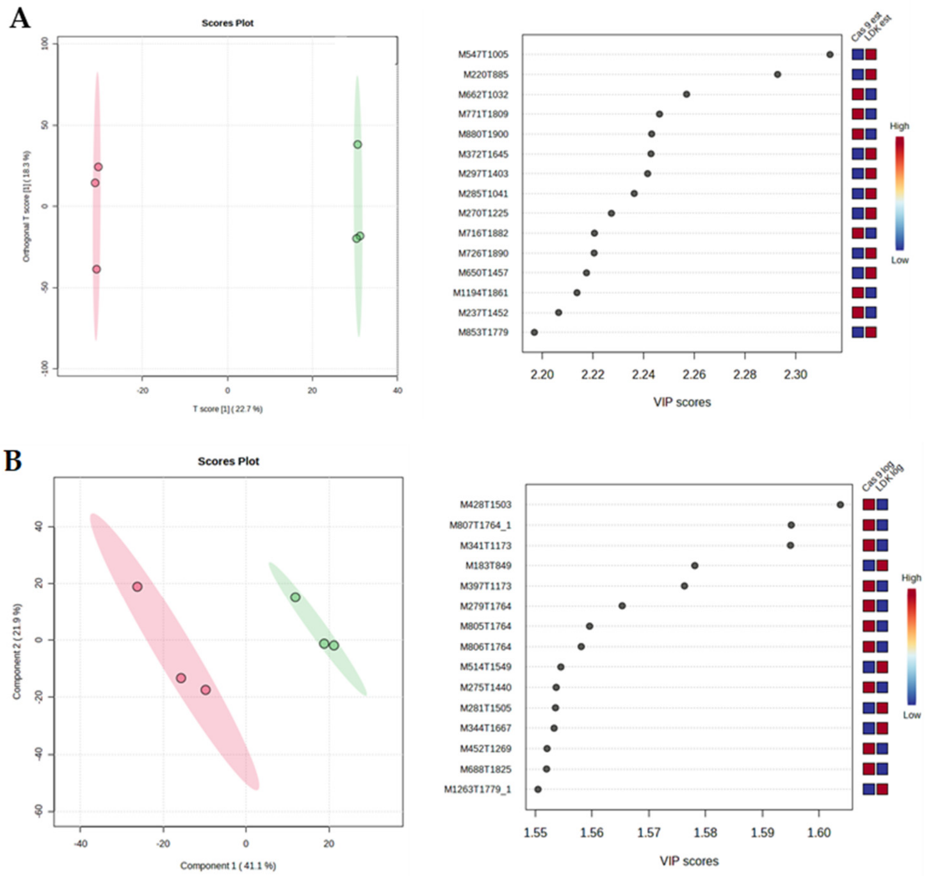

Figure S1. OPLS-DA and PLS-DA models for ΔLDK vs. Cas9. (**A**) OPLS-DA model for ΔLDK vs. Cas9 stationary phase (left) and top 15 respective VIP score (right), (**B**) PLS-DA model for ΔLDK vs. Cas9 logarithmic phase (left) and top 15 respective VIP score (right). Labels: red, Cas 9 and green, ΔLDK.

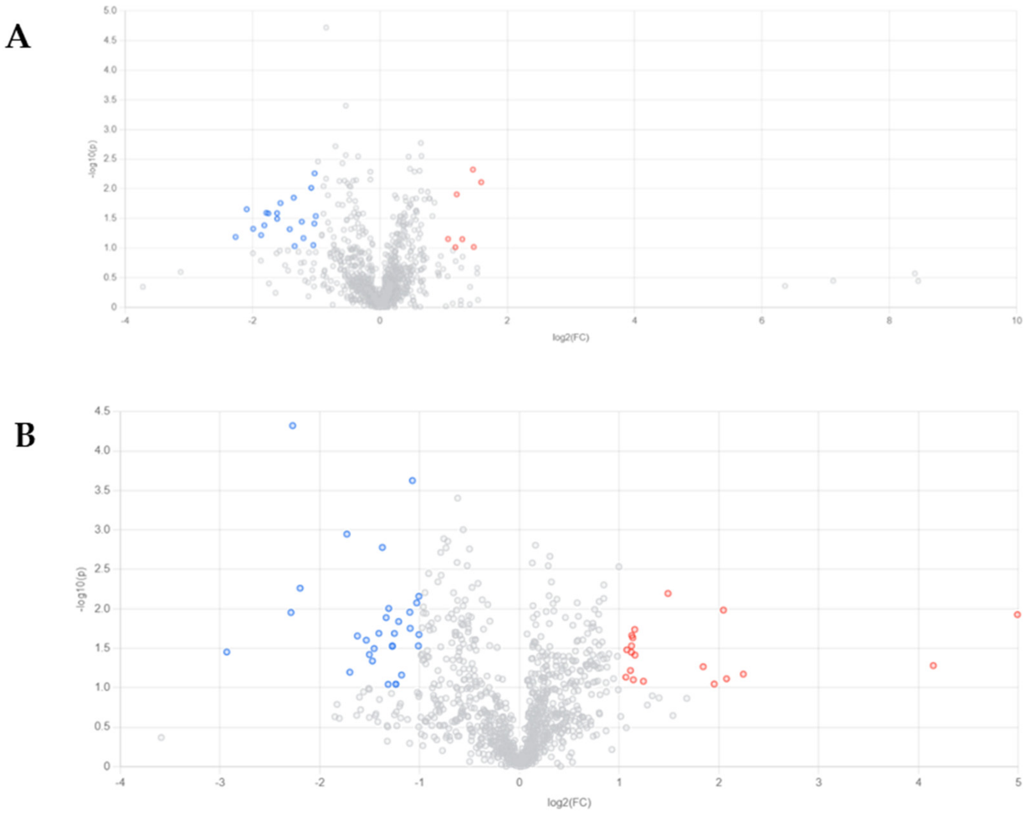

Figure S2. Volcano plots of molecular features altered in ΔLDK vs. Cas9 *L. infantum*. (**A**) Stationary phase and (**B**) logarithmic phase. Dots in blue and red indicate significantly decreased and increased, respectively, in ΔLDK compared to Cas9.

Table S1. Metabolite annotation and level of confidence according to the MSI (Metabolomics Standards Initiative).

Experimental mass	RT (min)	Adduct	m/z Error (ppm)	Molecular Weight	Name	Formula	Annotation MS/MS (Metaboscape - MoNa In Silico Spectra)	Annotation MS/MS (MSDial - MassBank library positive mode)	Annotation Level
560,5022	33,89	M+Na	2	537,5121	Cer(34:1;O2)	C34H67NO3	224.2370145807323 1040.0<br>225.24077424854394 228.0<br>236.23732478695337 10444.0<br>237.2405856004171 1480.0<br>254.24830649105442 1488.0<br>255.24910993044534 400.0<br>503.5040623198444 208.0<br>520.5095790521791 2136.0<br>521.5131362909655 700.0<br>522.5185548293151 236.0<br>538.5206650470718 196.0		2
395,3306	25,34	M+H	1	394,3236	Ergosta-5,7,22,24(28)-tetraen-	C28H42O	parent ion (LipidMaps)		2
482,324	24,72	M+H	0	481,3168	LPC(15:0)	C23H48NO7P		339.28973:208 341.30579:1060<br>342.30905:256 464.31702:268<br>465.31927:200 482.32504:1332	2
502,292	37,18	M+Na	3	479,3012	LPC(15:1)	C23H46NO7P	parent ion (LipidMaps)		2
516,3056	23,97	M+Na	1	493,3168	LPC(16:1)	C24H48NO7P	121.10022622574768 476.0<br>125.09481499624161 204.0<br>135.1163305415937 788.0<br>149.1320989552866 800.0<br>177.16467672155912 264.0<br>198.0540840347062 228.0<br>216.06179743208355 336.0<br>261.2569093779366 308.0<br>279.26904453273505 408.0<br>322.3102588703691 948.0<br>323.31577471059825 316.0<br>353.1361930863387 240.0<br>353.3055640339771 47356.0<br>353.3759758894968 192.0<br>354.3090804232376 10840.0<br>355.31188749857915 1204.0<br>356.3155083815379 208.0<br>476.31616206425485 480.0<br>477.31541941398353 284.0<br>494.32579298550706 8384.0<br>495.32913937852146 2312.0<br>496.3293706245388 516.0		2
524,371	24,79	M+H	0	523,3638	LPC(18:0)	C26H54NO7P	121.10022622574768 476.0<br>125.09481499624161 204.0	125.00024:1284 184.0733:13884<br>185.07617:796 258.11014:304	2
546,3526		M+Na	1						
522,3554	23,32	M+H	0	521,3481	LPC(18:1)	C26H52NO7P		125.00037:448 184.07329:7244<br>185.077:360 258.11099:192	2
544,3396		M+Na	4						
520,34	22,15	M+H	0	519,3325	LPC(18:2)	C26H50NO7P		124.9998:3552 126.00352:192<br>183.97081:196 184.03433:196<br>184.07338:44584 185.07576:2136<br>186.07797:288 337.27246:344<br>502.33057:352 520.3418:9920	2
518,3242	20,9	M+H	0	517,3168	LPC(18:3)	C26H48NO7P	124.99866546554992 1372.0<br>184.07307992304865 17964.0		2
540,3059		M+Na	0						
538,2899	20,47	M+Na	1	515,3012	LPC(18:4)	C26H46NO7P	124.99853723778497 528.0<br>184.07293255229456 5092.0<br>185.07649542234842 420.0<br>258.1089910979113 200.0<br>516.3089936058066 3408.0<br>517.3114478566368 852.0		2
568,3399	22,78	M+Na	5	545,3481	LPC(20:3)	C28H52NO7P	124.99847645573801 252.0<br>184.07304026020236 2436.0<br>546.3564019301674 980.0<br>547.3619409977473 380.0		3
544,3396	21,87	M+H	0	543,3325	LPC(20:4)	C28H50NO7P	124.99856544450155 228.0<br>184.0732285137866 3004.0<br>185.0762792032695 288.0<br>544.3399671905261 956.0<br>545.3446896879351 348.0		3
568,3399	22,11	M+H	0	567,3325	LPC(22:6)	C30H50NO7P	124.99883254415137 1520.0<br>184.07303027956877 19228.0<br>185.076650617497 768.0<br>258.10965445874496 268.0<br>550.3269825604298 464.0<br>551.336623202158 192.0<br>568.3411960355522 9876.0<br>569.3444793477785 3172.0<br>570.3475084788232 540.0		3

Experimental mass	RT (min)	Adduct	m/z Error (ppm)	Molecular Weight	Name	Formula	Annotation MS/MS (Metaboscape - MoNa In Silico Spectra)	Annotation MS/MS (MSDial - MassBank library positive mode)	Annotation Level
502,292	22,92	M+Na	3	479,3012	LPE(18:1)	C23H46NO7P	121.0999312845949 1812.0<br>122.10331861317326 344.0<br>123.11566411653945 444.0<br>124.01502130960209 244.0<br>125.09474077175271 380.0<br>135.11568626949926 3240.0<br>136.11909276367425 392.0<br>137.13151491474935 300.0<br>139.11122598598433 716.0<br>149.13172901433035 1904.0<br>150.13452460341705 208.0<br>151.1474987296985 580.0<br>153.1269663797367 728.0<br>163.1480389535018 824.0<br>165.16383623416567 568.0<br>167.14275705245709 492.0<br>177.1634644405975 636.0<br>178.16693208417823 220.0<br>179.1794001853839 248.0<br>181.15868614325828 388.0<br>191.1787345477779 424.0<br>195.17335453497907 252.0<br>198.05353660415867 592.0<br>205.19544081870373 252.0<br>216.06300630717428 888.0<br>247.24211565587854 1952.0<br>248.24580301729756 388.0<br>265.2522352225421 2708.0<br>266.2560237656811 504.0		2
478,2931<br>500,2746	21,77	M+H<br>M+Na	1<br>0	477,2855	LPE(18:2)	C23H44NO7P		119.08575:840 121.10168:1144<br>123.11652:1000 124.01505:192	2
337,2733	21,77	M+H	1	336,2664	methyl 5S,6R-epoxy-7-eicosynoate	C21H36O3	parent ion (LipidMaps)		2
277,2157	24,43	M+H	2	276,2089	Octadecatetraenoi	C18H28O2	parent ion (LipidMaps)		2
730,5393<br>752,5197	31,91	M+H<br>M+Na	2<br>1	729,5309	PC(32:2)	C40H76NO8P	124.99933624367908 748.0<br>184.07328854042953 21872.0		3
750,5057	30,64	M+Na	2	727,5152	PC(32:3)	C40H74NO8P	124.99883192424758 1496.0<br>183.71888964926387 200.0<br>184.0733107020476 63448.0<br>185.07639809734283 3448.0<br>186.07838225579812 424.0<br>294.31635214013056 192.0<br>307.2992249769766 456.0<br>337.27399217019087 2616.0<br>338.27883482534816 612.0<br>339.2896521931373 1116.0<br>340.2904077652764 200.0<br>353.30612860051025 200.0<br>392.2934986069773 928.0<br>393.29573220583575 200.0<br>587.5469562321139 216.0<br>728.5260968915348 6764.0<br>729.5304878681533 3116.0<br>730.5373061730811 724.0<br>730.5797785848454 540.0<br>731.5376415550422 192.0<br>731.5736177680294 268.0		3
738,5078<br>760,4868	31,03	M+H<br>M+Na	1<br>3	737,4996	PC(33:5)	C41H72NO8P	184.0727471398494 724.0<br>597.4878184274976 1660.0		3
780,5536	37,08	M+Na	3	757,5622	PC(34:2)	C42H80NO8P	124.99852129389825 804.0<br>184.07324613377992 39076.0<br>185.07652821524482 1384.0<br>186.07688463029763 224.0<br>756.5581590740636 344.0<br>757.5611045723526 388.0<br>758.5737277053532 2264.0<br>759.5778013692149 1204.0<br>760.5811538784554 288.0		3
756,5533<br>778,5394	32,38	M+H<br>M+Na	1<br>5	755,5465	PC(34:3)	C42H78NO8P	124.99856151656503 544.0<br>184.07303939368168 21544.0		3
754,538<br>776,5198	31,32	M+H<br>M+Na	0<br>0	753,5309	PC(34:4)	C42H76NO8P	124.99878975055614 692.0<br>184.073039124359 27496.0		3
750,5057	29,55	M+H	2	749,4996	PC(34:6)	C42H72NO8P	124.99936672942334 220.0<br>184.07297540411216 9600.0<br>185.07613065587222 400.0<br>413.2661079166052 664.0<br>414.27312334413807 232.0<br>750.5079524800087 876.0<br>751.5137110436658 296.0		3
786,6025<br>808,579	30,81	M+H<br>M+Na	2<br>5	785,5935	PC(36:2)	C44H84NO8P	124.99896099423715 1060.0<br>146.98092411369723 200.0		3

Experimental mass	RT (min)	Adduct	m/z Error (ppm)	Molecular Weight	Name	Formula	Annotation MS/MS (Metaboscape - MoNa In Silico Spectra)	Annotation MS/MS (MSDial - MassBank library positive mode)	Annotation Level
782,5685	32,33	M+H	1	781,5622	PC(36:4)	C44H80NO8P	124.99885745239766 2748.0		3
804,5514		M+Na	0				146.98041105381455 256.0		
780,5536	30,93	M+H	0	779,5465	PC(36:5)	C44H78NO8P	96.96882892279807 164.0		3
802,5354		M+Na	0				98.22513084792914 160.0		
778,5394	30,01	M+H	2	777,5309	PC(36:6)	C44H76NO8P	124.99921165338682 488.0		3
800,5209		M+Na	1				146.98108400681608 1772.0		
776,5218	29,53	M+H	1	775,5152	PC(36:7)	C44H74NO8P	124.99906309876886 2468.0		3
798,5038		M+Na	1				183.69537853780943 268.0		
832,5824	29,26	M+Na	0	809,5935	PC(38:4)	C46H84NO8P	124.9993509008029 840.0<br>146.9807103350844 984.0<br>184.07304676185305 29920.0<br>185.07635314016323 1512.0<br>186.07795058031715 232.0<br>624.5044655805317 256.0<br>807.5723891000439 584.0<br>808.585973028954 660.0<br>809.5951142190818 392.0<br>810.604426064883 2976.0<br>811.6080840370832 1552.0<br>812.6132583688548 596.0		3
806,5691	31,29	M+H	0	805,5622	PC(38:6)	C46H80NO8P	124.99864680311084 3636.0		3
828,5515		M+Na	0				146.98132918886878 852.0		
804,5514	32,17	M+H	3	803,5465	PC(38:7)	C46H78NO8P	124.9984822614908 428.0<br>146.98118832520436 3092.0<br>184.0731715637441 16940.0<br>185.0768454589059 624.0<br>463.22333548463143 244.0<br>465.2381987176456 232.0<br>599.5061183287754 500.0<br>621.4880242520188 1540.0<br>622.4918047371203 544.0<br>745.479961053224 824.0<br>746.4866607334866 336.0<br>802.5353751512135 224.0<br>804.5554106560213 4200.0<br>805.5585601698541 1628.0<br>806.5734276976395 2072.0<br>807.5764700757436 760.0<br>808.5861435690073 692.0		3
800,5209	30,23	M+H	2	799,5152	PC(38:9)	C46H74NO8P	146.9814329202542 2408.0		3
822,5062		M+Na	2				161.1301744925422 220.0		
836,6147	30,88	M+H	2	835,6091	PC(40:5)	C48H86NO8P	parent ion (LipidMaps)		3
830,5696	31,14	M+H	0	829,5622	PC(40:8)	C48H80NO8P	131.08438443913653 220.0		3
852,5523		M+Na	1				145.1015335283595 192.0		
828,5515	30,44	M+H	3	827,5465	PC(40:9)	C48H78NO8P	124.99885528621012 8800.0		3
850,5345		M+Na	1				126.00247122161426 336.0		
854,5666	30,88	M+H	3	853,5622	PC(42:10)	C50H80NO8P	parent ion (LipidMaps)		3
856,5813	31,47	M+H	4	855,5778	PC(42:9)	C50H82NO8P	124.99888247080821 588.0<br>146.98177367792033 248.0<br>184.07326069316304 45252.0<br>185.0763569784753 1828.0<br>186.0781451145019 316.0<br>856.5899504289667 3616.0<br>857.5937206659884 2056.0<br>858.5975827604225 632.0		3
880,5829	30,88	M+H	2	879,5778	PC(44:11)	C52H82NO8P	124.99865115376343 1808.0<br>183.68838999300542 228.0<br>183.7104502346088 308.0<br>183.81525197198602 228.0<br>183.85943949287608 224.0<br>183.94170572019655 308.0<br>183.9599029149316 388.0<br>183.9981732441444 276.0<br>184.07324795430532 103320.0<br>184.1239511733047 340.0<br>185.07673093795995 4856.0<br>186.07742394203822 524.0<br>878.5687775159385 340.0<br>879.5827672700455 344.0<br>880.5898699674715 9292.0<br>881.5938740563649 4884.0<br>882.596288777938 1452.0<br>883.6038660976694 336.0		3
286,3099	22,5	M+H	2	285,3032	Spisulosine	C18H39NO	parent ion (LipidMaps)		2
